# Evaluation of Online Patient Education Materials for Acute Myeloid Leukemia

**DOI:** 10.1007/s13187-025-02673-4

**Published:** 2025-06-26

**Authors:** Jessica M. Weiss, Yasmeen Mahayni, Amir S. Steinberg

**Affiliations:** 1 New York Medical College, Valhalla, NY USA; 2 Westchester Medical Center, Valhalla, NY USA

**Keywords:** Patient education materials, Acute myeloid leukemia, PEMAT

## Abstract

In the modern healthcare landscape, many patients turn to online platforms for information about their diagnoses and treatment options. This study assessed 14 acute myeloid leukemia (AML) websites for their understandability and actionability using the Patient Education Materials Assessment Tool (PEMAT). Fourteen patient-facing US AML websites were searched in Google, selected, and analyzed using the PEMAT criteria. PEMAT is an online resource used to evaluate print materials, with two sections: understandability and actionability. Websites were rated by two independent reviewers, and discrepancies were discussed. Websites were categorized by government, public/private companies, patient advocacy groups, professional societies, and cancer centers. The study analyzed 14 websites on AML education material. The mean “understandability” score was 86% (range 77%–100%); government scored the highest, and private/public companies scored the lowest. The mean “actionability” score was 63% (range 33%–100%); patient advocacy groups scored the highest, and cancer centers scored the lowest. Most of the AML websites analyzed were easy to understand but lacked material that prompted patients to take action regarding their care. These actions included writing down common questions to ask their physician and inquiring about resources (e.g., support groups, relevant clinical trials, and financial support). This study highlights an opportunity to enhance online patient education material by improving understandability through content summaries and visual aids and improving actionability by adding tools (e.g., planners, checklists) to help patients take control of their disease management, including comprehending treatment options, understanding disease etiology, and managing financial assistance.

## Background

In the current digital age, patients commonly turn to online health sources to educate themselves on their medical conditions. Nearly two-thirds of US adults said they searched the internet for health information within the last year. Of that group, 77% began their online query on search engines like Google, Bing, or Yahoo [1]. Patients with chronic conditions such as cancer are often highly engaged with their health via online information. A 2024 study assessing marginalized communities in Chicago showed that a majority of patients scheduled for an oncology visit used internet search engines to self-educate. When asked to select only one preferred health information source, 67% chose their doctor or health care provider, while 22% chose internet search engines [2].

The internet remains a critical educational source for cancer patients to learn more about their diagnoses, treatment options, and medication adverse effects. A 2018 report of phase I clinical trial patients found that most cancer-related internet use left patients feeling more empowered or relieved rather than anxious or confused [3]. Given the demand for digital cancer–related information, online sources must prioritize creating accurate, comprehensive, and easy-to-understand material for patients. This is especially important for older internet health-seekers who may have poorer digital literacy compared to younger populations. Studies have shown older patients reported greater difficulty in seeking health information online [4]. Groups who face challenges with online health-seeking were more likely to report negative perceptions about healthcare and be misinformed about cancer prevention [4, 5].

Our study aimed to assess and compare the quality of various patient-facing acute myeloid leukemia (AML) online sources. Researchers have previously conducted similar studies on other diseases such as attention-deficit/hyperactivity disorder, atrial fibrillation, and bladder cancer [6–8].We chose to explore AML because of its significant impact on older adults aged 60–70; it accounts for approximately 80% of all cases of adult leukemia.

## Methods

### Data Search and Selection

Fourteen patient-facing US online AML websites were found via Google, categorized, and evaluated using the Patient Education Materials Assessment Tool (PEMAT) [9]. Our inclusion criteria were websites that were geared towards patients and explained AML symptoms, management, and treatment. Exclusion criteria were websites that specifically targeted healthcare providers or were unrelated to AML. The sample size of 14 websites was chosen as a feasible and representative number to capture the most popular sources across multiple categories. The websites evaluated were National Cancer Institute (NCI), MedlinePlus, American Society of Hematology (ASH), American Society of Clinical Oncology (ASCO), American Cancer Society, Leukemia and Lymphoma Society, WebMD, Healthline, Medical News Today, and 5 National Comprehensive Cancer Network (NCCN) designated cancer centers. The websites were classified as government, professional societies, patient advocacy groups, public/private companies, and cancer centers.

### Assessment Tool

The PEMAT rating tool is a validated and freely accessible online resource used to evaluate print and audiovisual information. The Patient Education Materials Assessment Tool for Printable Materials (PEMAT-P) includes an “understandability” section, with 17 items, and an “actionability” section, with 6 items [9]. “Understandability” is the degree to which the material allows readers to process and comprehend key points. “Actionability” describes the extent to which websites successfully identify actions readers can take towards their health. Each item is rated as either agree (1 point), disagree (0 points), or not applicable (no points allocated) [9]. Two independent reviewers scored each website, and any discrepancies were discussed. Both reviewers are proficient in the English language, categorized at the level C2, based on the Common European Framework of Reference for Languages [10].

Final scores were calculated as a percentage of “agree” responses divided by the total possible points, excluding “not applicable” items. Websites were considered understandable and actionable if they reached a score of ≥70% on both metrics, as has been previously validated [11].

### Ethics Approval

This study focused on the quality assessment of publicly available websites; ethics committee approval was unnecessary.

## Results

Of the websites assessed, all 14 met the benchmark for understandable, while only four (29%) met the benchmark for actionable. The mean PEMAT-P understandability score was 86% (*range*: 77%–100%) and the mean actionability score was 63% (*range*: 33%–100%). One (7%) website scored 100% in understandability and two (14%) websites scored 100% in actionability. The 14 websites were then categorized based on type of organization and scores were analyzed accordingly (Table 1). Based on understandability, government websites scored the highest on average, followed by patient advocacy groups, professional societies, cancer centers, and private/public companies. As for actionability, patient advocacy groups scored the highest, followed by professional societies, government, private/public companies, and cancer centers (Table 1).

**Table 1:** Average, minimum, and maximum understandability and actionability scores for each website category

Website Category	Understandability score (%)			Actionability score (%)		
	Mean	Minimum	Maximum	Mean	Minimum	Maximum
Cancer centers (*n *= 5)	85.0%	76.9%	88.2%	50.7%	33.3%	80.0%
Government (*n *= 2)	92.3%	84.6%	100.0%	63.3%	60.0%	66.7%
Patient advocacy groups (*n *= 2)	89.9%	85.7%	94.1%	90.0%	80.0%	100.0%
Private/public companies (*n *= 3)	80.5%	76.5%	88.2%	53.33%	50.00%	60.00%
Professional societies (*n *= 2)	86.1%	84.6%	87.5%	80.0%	60.0%	100.0%
Weighted mean	85.9%	76.5%	100.0%	62.9%	33.3%	100.0%

**Fig. 1:**
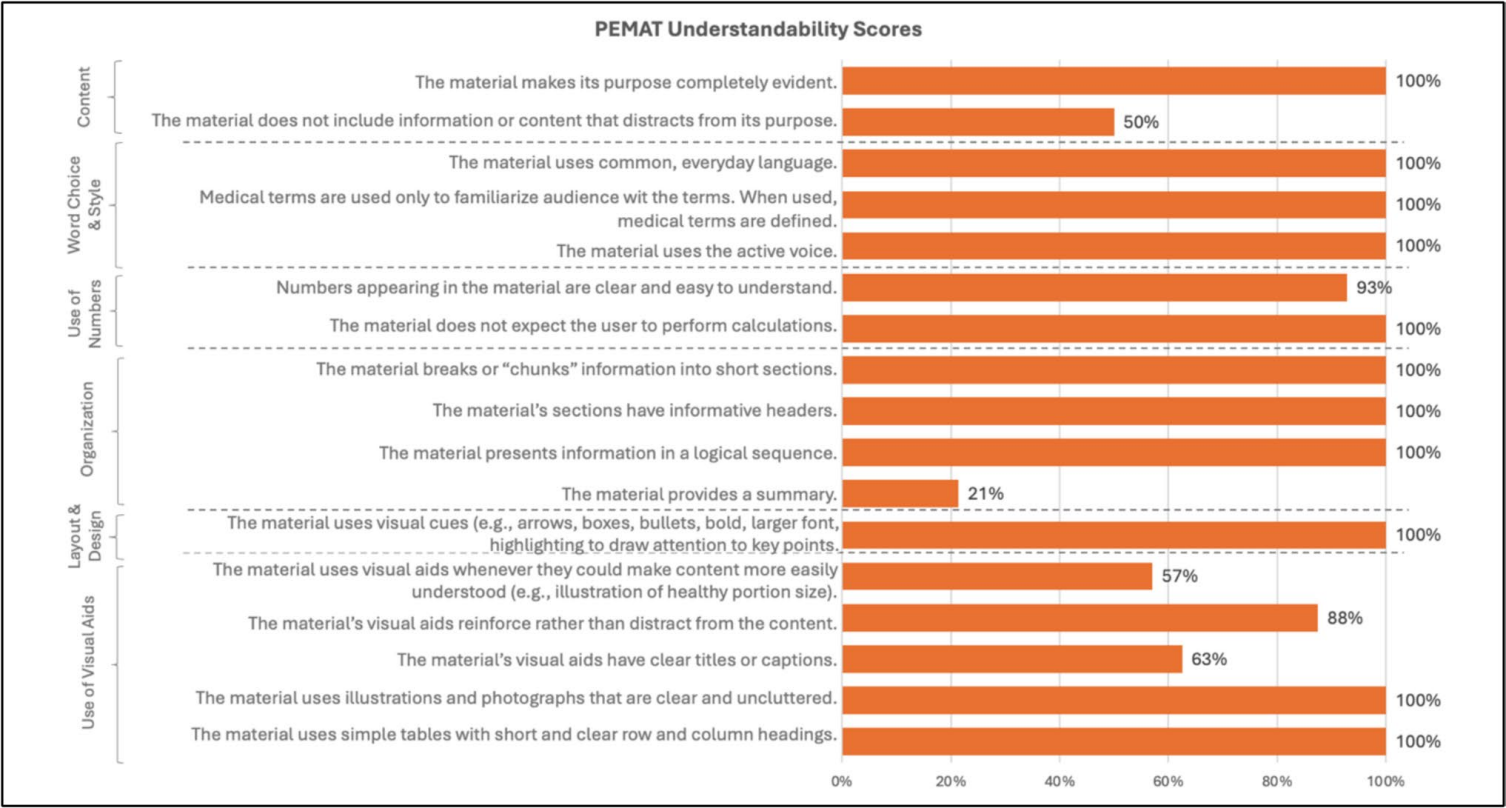
Patient Education Materials Assessment Tool for Printable Materials scoring. Percentage of websites that scored “agree” for each understandability item

Most websites scored fairly well in understandability (Figure 1); however, the lowest scoring domains included providing a summary of the material and implementing visual aids. Cancer centers and private/public companies typically scored lower in understandability due to content that distracted from the website’s purpose, such as the inclusion of advertisements and marketing chatbots.

**Fig. 2:**
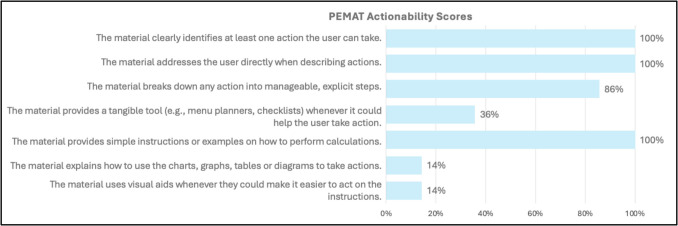
Patient Education Materials Assessment Tool for Printable Materials scoring. Percentage of websites that scored “agree” for each actionability item

In terms of actionability, all of the websites successfully identified the reader and outlined at least one action they can take towards their health; of those websites, 86% break down the action into manageable, elicit steps. The lowest scoring actionability domains centered around including and explaining visual aids to help users act on instructions. Only five sources (36%) provided a tangible tool (menu planners, checklists) to help take action (Figure 2).

## Discussion

The percentage of patients who seek answers to their medical questions online has risen over the last decade. In 2008, 61% of people searched the internet first for health information; by 2017, this number increased to 74% [4]. As digitally literate generations age and navigate the healthcare system, online health content will become more and more relevant. We must ensure that online education sources provide accurate, digestible, and empowering information so patients can engage with their health without anxiety or confusion.

### Suggestions for Future Education Materials

**Fig. 3:**
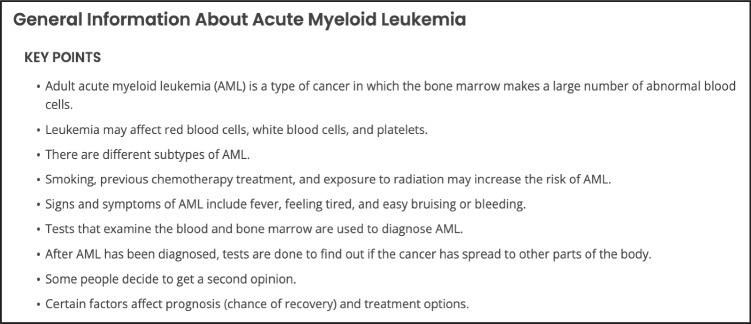
Example of effective content summary [12]

Based on the average PEMAT scores, we provide suggestions to improve understandability and actionability. The first suggestion to improve understandability is incorporating content summaries into each website. Only 21% of the assessed education materials contained a summary, which is useful to emphasize salient points and distill long or complex topics. According to the PEMAT‐P guidelines, summaries can appear at the beginning or the end of content and can also be formatted as “key steps” for the patient to take. Figure 3 shows an example of an effective summary taken from one of the Government sources on AML. It communicates key points about the disease overview, symptoms, diagnostic tests, and treatments.

**Fig. 4:**
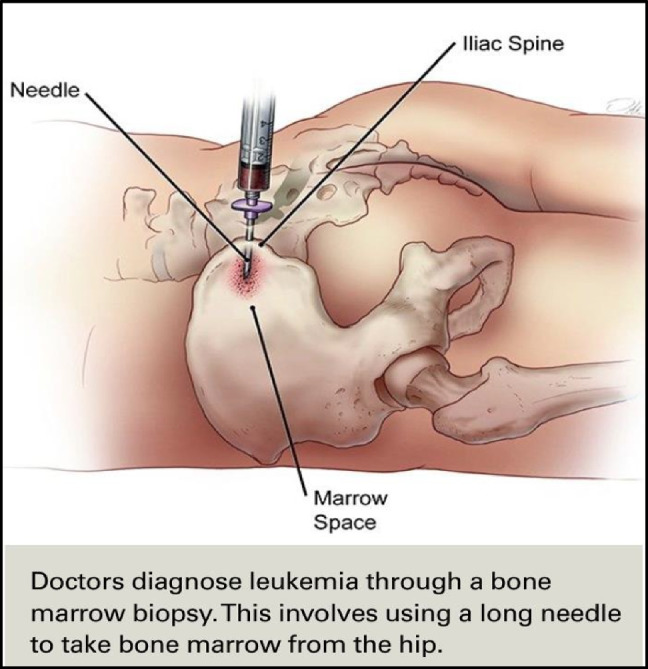
Example of easily understood visual aid with appropriate caption [13]

Another suggestion to improve the understanding of patient-friendly education material is the use of visual aids. PEMAT-P guidelines state that a graphic or illustration should be used whenever it can elucidate a topic. Only 57% of the sources included visual aids, and of those, only 63% had visuals accompanied by clear titles or captions. When rating, if a website did not contain any visual aids, it was assigned “N/A”; if a source contained misleading pictures (e.g., generic, unrelated stock photos), it received a rating of “0.” Graphics should reinforce the content rather than distract from it; for example, Figure 4 is taken from a cancer center’s website and helps demonstrate a bone marrow biopsy with both an illustration and a succinct caption.

**Fig. 5:**
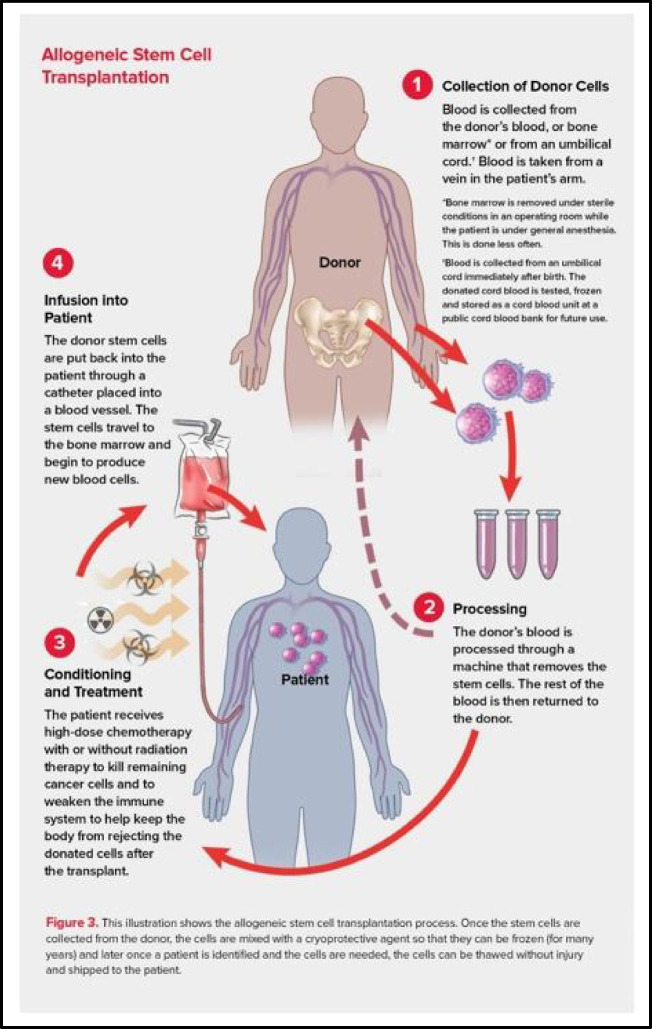
Example of a clear visual aid explaining allogenic stem cell transplant procedure for AML [14]

Other helpful areas to incorporate visual aids may be in the disease overview and the therapeutic modalities section. Figure 5, taken from a Patient Advocacy Group website, successfully conveys how an allogenic stem cell transplant is performed, a common procedure for patients with AML. We suggest keeping drawings simple and minimizing scientific jargon to accommodate all health literacy levels.

**Fig. 6:**
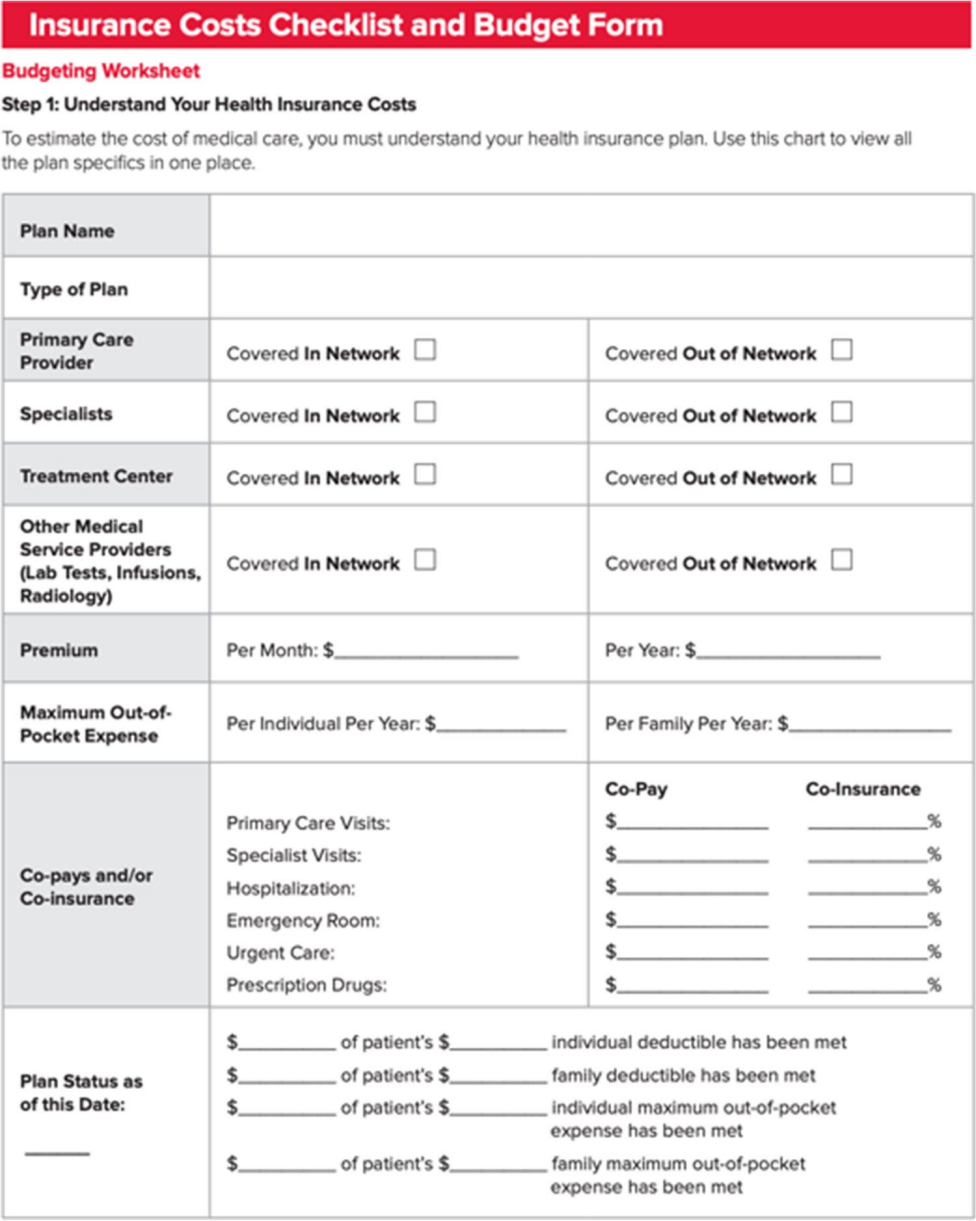
Example of a financial resources tool to help patients manage their medical care costs [15]

**Fig. 7:**
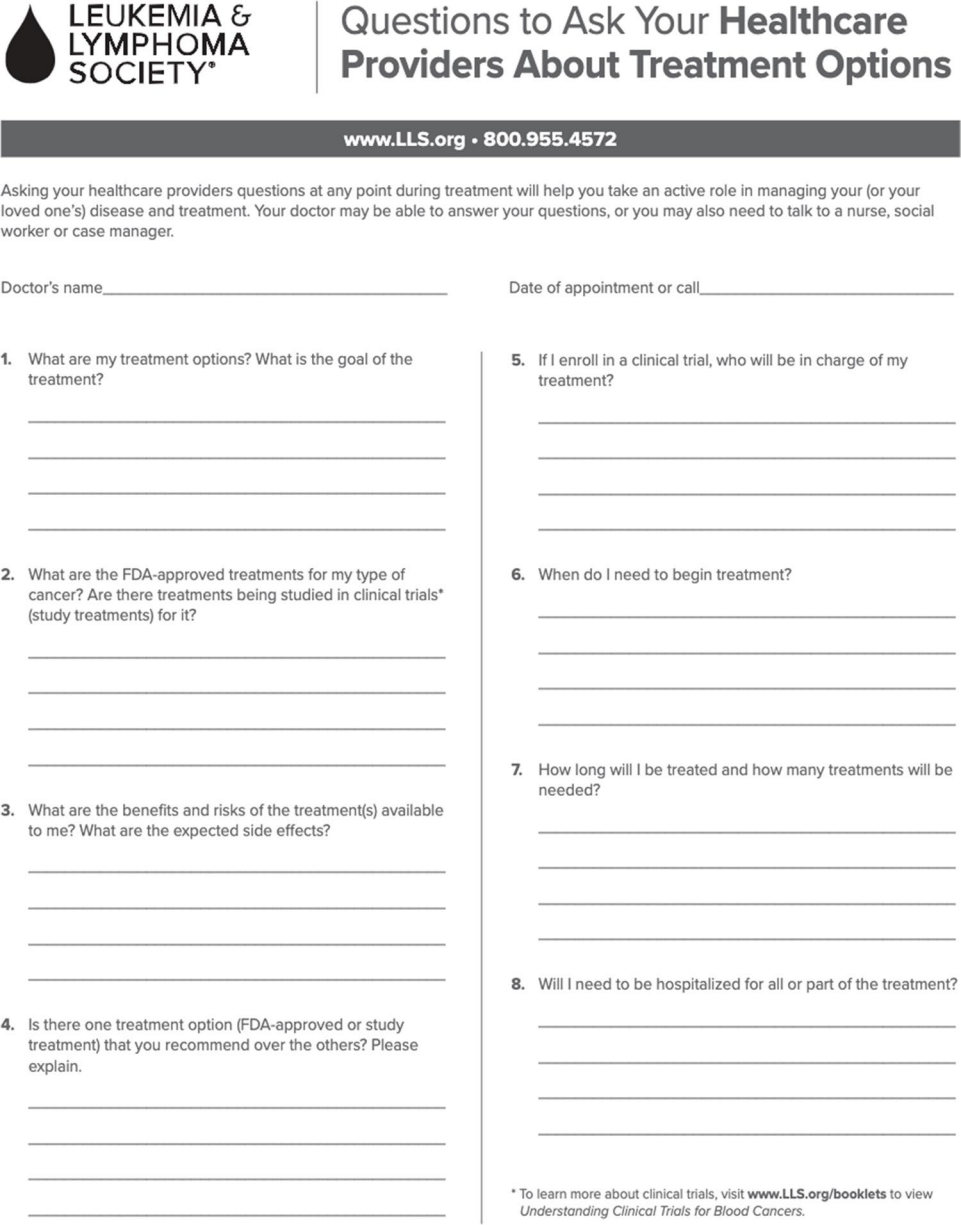
Example of a worksheet to empower patients to inquire about treatment options [16]

In terms of improving the actionability of patient education websites, we suggest adding action-oriented features in the form of tangible health tools. These tools are intended to help patients take control of their disease management, including—but not limited to—comprehending the treatment options available to them, understanding the etiology and prognosis of their disease, and managing financial support options. For example, patients can be prompted to write down common questions to ask their physician about their disease and inquire about resources available to them (e.g., support groups, relevant clinical trials, and financial support)*.* We found that only 36% of the websites evaluated used tangible tools to help the user take action. Figures 6 and 7 are examples of practical resources that help patients seek further information about financial resources and treatment options. These tools allow patients to remain engaged with their health and take a proactive approach in health management.

#### Strengths and Limitations of the Study

A strength of this study was its standardized procedure that used two independent reviewers to evaluate and rate the websites. In addition, a clinically validated tool, PEMAT, was used to systematically assess each source. Although the evaluated websites spanned multiple categories, such as government websites, professional societies, patient advocacy groups, public/private companies, and cancer centers, the sample size of evaluated patient education websites was small. Further, the sources were limited to English-language, US-based websites, possibly introducing bias due to the lack of information on the ethnic and racial backgrounds of patients. Given the US’s large immigrant population, ideally the websites should take into account cultural differences and provide further language options. We propose future research evaluating the educational content in other countries and recruiting colleagues for assistance in exploring non-English language material.

Lastly, the PEMAT-P criteria is intended to be used by healthcare personnel involved in patient education. Since the raters in our study were medical students, this may introduce bias due to differing health literacy, socioeconomic, and educational profiles compared to the average patient.

## Conclusion

As many patients turn to online website sources to understand their health, it is crucial that educational materials provide quality information that patients can appropriately understand and act on. This study highlights opportunities to enhance online patient education materials by adding features to enhance understandability, such as content summaries and visual aids. It also emphasizes adding features to enhance actionability such as tangible tools (e.g., menus, planners, checklists) to help patients better grasp their disease, in terms of treatment options, etiology and prognosis, and financial support.
